# Stat-6 signaling pathway and not Interleukin-1 mediates multi-walled carbon nanotube-induced lung fibrosis in mice: insights from an adverse outcome pathway framework

**DOI:** 10.1186/s12989-017-0218-0

**Published:** 2017-09-13

**Authors:** Jake Nikota, Allyson Banville, Laura Rose Goodwin, Dongmei Wu, Andrew Williams, Carole Lynn Yauk, Håkan Wallin, Ulla Vogel, Sabina Halappanavar

**Affiliations:** 1 Environmental Health Science and Research Bureau, Health Canada, Ottawa, ON K1A 0K9 Canada; 2 Department of Biological and Chemical Work Environment, National Institute of Occupational Health, Oslo, Norway; 3 National Research Centre for the Working Environment, Lerso Parkallé 105, DK-2100 Copenhagen, Denmark; 4 Department of Micro- and Nanotechnology, Technical University of Denmark, DK-2800 Kgs. Lyngby, Denmark

**Keywords:** Nanomaterials, Multi-walled carbon nanotubes, Inflammation, Fibrosis, Lung disease, Il-1, STAT6, Adverse outcome pathway, M2 Macrophage

## Abstract

**Background:**

The accumulation of MWCNTs in the lung environment leads to inflammation and the development of disease similar to pulmonary fibrosis in rodents. Adverse Outcome Pathways (AOPs) are a framework for defining and organizing the key events that comprise the biological changes leading to undesirable events. A putative AOP has been developed describing MWCNT-induced pulmonary fibrosis; inflammation and the subsequent healing response induced by inflammatory mechanisms have been implicated in disease progression.

The objective of the present study was to address a key data gap in this AOP: empirical data supporting the essentiality of pulmonary inflammation as a key event prior to fibrosis. Specifically, Interleukin-1 Receptor1 (IL-1R1) and Signal Transducer and Activator of Transcription 6 (STAT6) knock-out (KO) mice were employed to target inflammation and the subsequent healing response using MWCNTs as a model pro-fibrotic stressor to determine whether this altered the development of fibrosis.

**Results:**

Wild type (WT) C57BL/6, IL-1R1 (KO) or STAT6 KO mice were exposed to a high dose of Mitsui-7 MWCNT by intratracheal administration. Inflammation was assessed 24 h and 28 days post MWCNT administration, and fibrotic lesion development was assessed 28 days post MWCNT administration. MWCNT-induced acute inflammation was suppressed in IL-1R1 KO mice at the 24 h time point relative to WT mice, but this suppression was not observed 28 days post exposure, and IL-1R1 KO did not alter fibrotic disease development. In contrast, STAT6 KO mice exhibited suppressed acute inflammation and attenuated fibrotic disease in response to MWCNT administration compared to STAT6 WT mice. Whole genome analysis of all post-exposure time points identified a subset of differentially expressed genes associated with fibrosis in both KO mice compared to WT mice.

**Conclusion:**

The findings support the essentiality of STAT6-mediated signaling in the development of MWCNT-induced fibrotic disease. The IL-1R1 KO results also highlight the nature of the inflammatory response associated with MWCNT exposure, and indicate a system with multiple redundancies. These data add to the evidence supporting an existing AOP, and will be useful in designing screening strategies that could be used by regulatory agencies to distinguish between MWCNTs of varying toxicity.

**Electronic supplementary material:**

The online version of this article (10.1186/s12989-017-0218-0) contains supplementary material, which is available to authorized users.

## Background

Carbon nanotubes are among the widely produced nanomaterials (NMs) globally [1]. Multi-walled carbon nanotubes (MWCNTs) are the most used variants of this NM class with a growing number of commercial and industrial applications [2]. The diverse applications of MWCNTs are attributed to their unique physical-chemical properties. MWCNTs possess a fiber-like structure with a diameter of up to 100 nm and lengths up to 28,000,000 times their diameter [2], because of which they exhibit exceptional benefits such as high mechanical strength, stiffness, and superior thermal and electric conductivity properties. Moreover, MWCNTs are polymers of carbon and therefore are amenable for manipulation of their surface structure. Chemical modifications of MWCNTs can aid in better solubility and dispersion of the material for various applications. The very unique and commercially attractive properties also render them toxic, which is a major issue [3]. The high-aspect ratio of MWCNTs is comparable to other high aspect ratio substances such as asbestos, raising further concerns about their use in various applications [4, 5]. Complicating the situation is the fact that there are many variants of MWCNTs exhibiting distinct properties that are suggested to uniquely influence the toxicological outcomes induced by these materials. Thus, there is a pressing need to characterize the toxicity induced by these materials and the underlying mechanisms associated with this toxicity. More urgently, strategies and tools to rapidly screen toxicity of different types of MWCNTs and predictive markers of exposure and effects of MWCNTs are needed.

Several studies have shown that when inhaled, MWCNTs persist in lungs and induce injury leading to interstitial and sub-pleural lung fibrosis and granulomas in rodents. Recent literature suggests that the hallmarks of MWCNT-induced fibrotic response involve an acute inflammatory response that is predominantly neutrophilic in nature, chronicity of inflammation, and ultimately clinical manifestation of fibrotic lesions [6]. However, the essentiality of acute lung inflammation in lung fibrosis induced by MWCNTs is unclear. Lung fibrosis is induced following repeated exposure to certain types of bacteria or viruses as well as following repeated exposure to toxic chemical substances. Studies involving targeted inhibition of specific inflammatory mediators have demonstrated that inflammatory processes play a role in the underlying mechanisms of fibrosis induced by these pro-fibrotic stimulants [7, 8]. For example, targeting inflammatory mediators such as IL-17A reduced the number of fibrotic lesions in mice exposed to the lung-damaging peptide bleomycin or fibrosis-inducing bacteria [9, 10]. Similarly, blocking the classical mediator of inflammation, Tumor necrosis factor (TNF)-α, resulted in decreased fibro-proliferative disease in the lungs of mice exposed to asbestos [11], and targeting reactive oxygen species (ROS) synthesis accompanying an inflammatory response resulted in decreased incidences of asbestos-induced fibrotic lesions [12]. The inflammatory response is a crucial initiator of secretion of growth factors and activation of T helper (Th) 2 type cells that are known to drive the healing response, uncontrolled activation of which is implicated in the development of fibrosis [9, 10]. Relatively few inflammatory mediators and genes associated with the healing response have been specifically studied to determine their role in response to MWCNT-exposure and pathogenesis. Such data would provide crucial insight into the key events that lead to MWCNT-induced disease and help identify sensitive markers of exposure and adverse effects of MWCNTs.

**Fig. 1:**
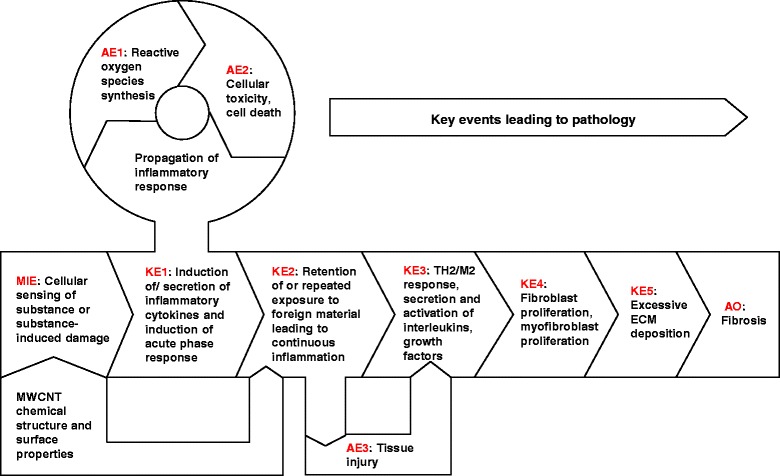
Adverse Outcome Pathway (AOP) for the development of MWCNT-induced lung fibrosis. Graphical depiction of the key events that describe the progression from inhalation exposure to MWCNTs to the development of pulmonary fibrosis. The figure highlights the molecular initiating event (MIE), key events (KE), associative events (AE), and the adverse outcome (AO)

Using MWCNT as a prototype stressor, we recently published a putative Adverse Outcome Pathway (AOP) for lung fibrosis, and identified a Molecular Initiating Event (MIE) and Key Events (KEs) potentially involved in the pathology (Fig. 1) [13]. In brief, the MIE involves cellular sensing of the MWCNTs in the lungs and the release of danger signals, leading to activation of KE1 - induction of inflammatory cytokines/chemokines/growth factors leading to activation of KE2. This is also associated with infiltration of inflammatory cells into the lung tissue and is characterized as acute inflammation. KE2 involves retention of MWCNTs, which is associated with the persistence of inflammatory signals, synthesis of reactive oxygen species and lung injury, all acting in a positive feedback loop leading to KE3. KE3 marks deregulated wound healing process, which is measured as activation of Th2 type cells and M2 type macrophages, and secretion of anti-inflammatory mediators and growth factors that play an important role in the progression of lung fibrosis. KE4 and KE5 involve activation of fibroblast/myofibroblast proliferation and uncontrolled ECM deposition leading to fibrotic lesions in the lungs. In essence, this AOP hypothesized that acute and subsequent chronic inflammatory conditions play a role in MWCNT-induced lung fibrosis. In the present study, we used knock out models of an inflammatory pathway and the wound healing response to specifically inhibit inflammation or the subsequent healing response as defined in the AOP to investigate their essentiality to the overall pathogenesis of lung fibrosis induced by MWCNTs. Specifically knocking out or enhancing KEs to demonstrate expected impacts on downstream KEs and the AO in the expected direction is one of the main elements of weight of evidence (criteria: essentiality) assessment in support of an AOP [14, 15]. In this study, we used knock-out mice to specifically investigate the essentiality of Interleukin (IL)-1 signaling pathway to inhibit acute inflammatory response (KE1) and Signal Transducer and Activator of Transcription 6 (STAT6) mediated signaling (KE3).

The IL-1 signaling pathway is a key coordinator of inflammation induced by exposure to various inhaled toxicants [16, 17]. IL-1 was one of the first cytokines to have been characterized, and its signaling is accomplished through the binding of the IL-1 receptor (IL-1R1) to one of two ligands, IL-1α or IL-1β [18]. IL-1 signaling has been implicated in the development of pulmonary fibrosis; overexpression of IL-1β in the lungs of mice results in the development of fibrotic lesions [19]. This is further supported by the observations that disruption of IL-1 signaling results in less fibrosis in IL-1R1 and MyD88 deficient mice following treatment with bleomycin [20]. More recently, it was shown that MWCNT-induced lung inflammation is mediated by IL-1 signaling [21], but a complete characterization of how the biological responses to MWCNTs are impacted in the absence of IL-1 signaling and its repercussions on fibrotic pathology has not been assessed. The transcription factor STAT6 is a crucial mediator of Th2 responses [22, 23]. Experimental models of fibrosis have also found less disease in STAT6 deficient mice [24, 25]. STAT6 phosphorylation has been measured after MWCNT exposure and it has been proposed to be involved in the development of MWCNT-induced lung pathology [26], yet STAT6 has not been directly targeted in a model of MWCNT exposure.

In the current study we investigated the essentiality of IL-1 and STAT6 signaling-mediated lung inflammation in MWCNT-induced lung fibrosis. We exposed IL-1R1 deficient mice or STAT6 deficient mice to Mitsui XNRi-7 (Mitsui-7), a MWCNT variant known to induce lung fibrosis in experimental rodents. Detailed histopathology and global gene expression analysis was performed to characterize the lung responses 1 and 28 day post-intratracheal instillation of 162 μg/mouse dose of Mitsui-7 and to assess impacts of the KO on downstream KE and the AO, for pulmonary fibrosis.

## Methods

### MWCNT characteristics

This study utilized Mitsui XNRi-7 (lot 05072001 K28, Hodoga Chemical Industry (formerly known as Mitsui)), which has been classified as a possible human carcinogen (Group 2B) [27]. Physical-chemical characteristics of Mitsui-7 have been published previously [28]. In brief, Mitsui-7 are described as rod-like fibers with an average length of 3.86 μm and diameter of 49 ± 13.4 nm [28]. Collision type Inductively Coupled Plasma Mass Spectrometry, Combustion Ion Chromatography and trace metal analysis detected some impurities, which included Fe: 0.3%, Na: 0.4%, S: ca. 470 ppm and Cl: ca. 20 ppm. This batch of Mitsui-7 has been assessed for endotoxin contamination previously and is shown to contain negligible levels of endotoxin [29].

### MWCNT preparation and administration

Mitsui-7 suspensions were prepared fresh for each experiment. A total of 8.9 mg of Mitsui-7 was suspended in NanoPure water containing 2% serum collected from C57BL/6 mice to a total stock suspension of 3.24 mg/ml. Suspensions were prepared by sonicating the particle preparations using a Branson Sonifier S-450D (Branson Ultrasonics Corp., Danbury, CT) equipped with a disruptor horn (Model number: 101–147-037). Total sonication time was 21 min at 40 W. The samples were continuously cooled on ice during the sonication procedure. 50 μl of the suspension was used for the 162 μg/mouse dose. Vehicle controls were prepared as described above with only NanoPure water and 2% serum. The 162 μg/mouse dose was selected based on the results of our previous studies, at which Mitsui-7 is shown to induce fibrosis in C57BL/6 mice [30]. Mitsui-7 was specifically chosen because of the established observation of lung pathology induced by this MWCNT [30, 31].

### Animal care and exposure

Female wild type C57BL/6 mice (WT) and C57BL/6 mice deficient in IL-1R1 (IL1-R1 KO) or STAT6 (STAT6 KO), age 5–8 weeks old, were purchased from Jackson Laboratory (Bar Harbor, ME). Mice were acclimatized for a week. Mice were housed under specific pathogen-free conditions on a 12-h light-dark cycle with food and water provided ad libitum. All animal procedures were approved and followed the care and handling guidelines for laboratory animals established by the Health Canada Animal Care Committee.

### Animal exposure and tissue collection

Each treatment group consisted of 10 animals; 5 animals were used to collect tissues for bronchoalveolar lavage fluid (BAL) and RNA/protein extraction, and 5 animals were used to collect tissues for histology. Mice were anesthetized by inhalation of 5% isoflurane (Isoflo, Esteve Farma, Carnaxide, Portugal) in 100% oxygen. Mice in the experimental group received a single intratracheal instillation of 162 μg of Mitsui-7 in a 50 μl freshly prepared suspension, as described above, followed by 150 μl of air with a 250 μl SGE glass syringe (250F–LT-GT, MicroLab, Aarhus, Denmark). Control mice received 50 μl of vehicle (2% serum in nanopure water) only followed by 150 μl of air. Mice were kept under observation until they recovered from anesthesia. Mice were sacrificed 1 and days 28 post-exposure. BAL and lung tissue were collected. BAL was performed by lavaging the lungs twice with 1 ml saline using a 2 ml syringe. Each lavage consisted of 3 up and down movements performed slowly (5–10 s each) and the fluid was immediately placed on ice until the analysis. Post-lavage, the right and left lobes of lung were cut in pieces, snap frozen in liquid nitrogen, and stored at −80 °C. The lung tissues from other 5 animals were fixed for histology as described below under the histology section.

### BAL cell count

The combined lavage volume recovered was estimated and BAL fluid and BAL cells were separated by centrifugation at 4 °C and 400 g for 10 min. The total cell number (TCN) was determined per volume using a Moxi Z OS 4.0 cell counter (ORFLO Technologies, Hailey, ID). Cytospins were prepared and stained with Hema 3 (Bio- chemical Sciences, Swedesboro, NJ). Five hundred cells were counted per cytospin for determination of percent mononuclear cells and percent neutrophils. Differential cell counts were calculated using this percentage and the total cell number.

### BAL inflammatory cytokine measurement

IL-1α, CXCL1, IL-6, IL-12p40, CCL2, IL-5, and granulocyte colony stimulating factor (G-CSF) were measured in BAL by Mouse Cytokine Bio-Plex Pro (Bio-Rad, Hercules, CA). IL-1β, osteopontin (OPN), and transforming growth factor (TGF)-β were measured in BAL by Quantikine ELISA (R&D Systems, Minneapolis, MN). All assays were conducted as specified by the manufacturer’s instructions.

### Histology

Lungs were uniformly fixed at 30 cm H_2_O pressure in 10% formalin for histological assessment. After a minimum of 24 h formalin fixation, lungs were paraffin embedded, and 4-mm thick slices were cut. The resulting slides were stained with hematoxylin and eosin (H&E) to measure pathological changes and Masson Trichrome stain to assess collagen deposition. Additionally, immunohistochemistry for the fibroblast marker vimentin (Rabbit vimentin antibody, Catalogue #5741, Clone D21H3, 1:100 dilutions; Cell Signaling Technologies, Danvers, MA) was also performed on formalin-fixed, paraffin embedded lung tissue sections.

For the quantification of fibrotic lesions, the entire longitudinal cross-section of the lungs was captured in a series of images from H&E stained lung sections. From these images the total cross-sectional area was traced and measured using ImageJ software (National Institute of Health, Bethesda, MD). The disease area was defined by thickened alveolar septa, which corresponded with areas of collagen deposition as determined by the Masson Trichrome staining. The disease area was traced with ImageJ software and expressed as a percentage of the total cross-sectional area. These measurements were performed by two independent researchers.

### Hyperspectral microscopy

Mitsui-7 fibers were visualized by hyperspectral imaging as previously described [32]. Hyperspectral images were taken of H&E stained histology samples using a CytoViva nanoscale hyperspectral microscope (Cytoviva, Inc., Auburn, AL, USA). This imaging system integrates a visible and near-infrared (VNIR) spectrophotometer (400–1000 nm), a Dage Excel Color Cooled-M camera, and an Olympus BX 43 optical microscope. Image acquisition was taken at 100× magnification and analysis was carried out with Environment for Visualization (ENVI 4.8, Cytoviva, Inc. Auburn, AL, USA) software. Prior to analysis of samples, a reference spectral library was created for Mitsui-7. Spectra from Mitsui-7-exposed samples were compared to this reference library by Spectral Angle Mapping, a spectral classification algorithm in ENVI that used an n-D angle to match pixels from the treated samples to reference spectra. Spectral similarity is established between two spectra by calculating the angle between them and converting them to vectors in a space with dimensionality equal to the number of bands. Pixels further away than the maximum angle (radians) threshold for spectral classification of 0.1 were not classified.

### Collagen and total protein quantification in BAL

Soluble collagen was quantified in BAL fluid by Sircol assay (Biocolor Life Science Assays, Carrickfergus, UK). Total protein as an indication of proteinosis was measured in BAL by Bradford Assay (Bio Rad, Hercules, UK).

### RNA isolation and purification

A small random section of the snap frozen right lung was homogenized immediately in TRIzol reagent (Invitrogen, Carlsbad, CA, USA) using the Retsch Mixer MM 400. The RNA was isolated using chloroform and precipitated using isopropyl alcohol. The RNA was subsequently purified using RNeasy Mini Plus kits (Qiagen, Mississauga, ON, Canada). Integrity of the RNA samples was analyzed using an Agilent 2100 Bioanalyzer (Agilent Technologies, Mississauga, ON, Canada). All samples had an RNA integrity number above 5.5 and were all used for microarray analysis.

### Microarray hybridization and statistical analysis of microarray data

For each individual lung tissue sample (*n* = 5/treatment group) and Universal Mouse Reference RNA (UMRR, Stratagene, Mississauga, ON, Canada), 200 ng of total RNA was used to synthesize cDNA and cyanine-labeled cRNA (the experimental samples were labeled with cyanine-5 and cyanine-3 was used to label the reference RNA) using the Agilent Linear Amplification Kit (Agilent Technologies, Mississauga, ON, Canada). Labeled cRNA was transcribed using T7 RNA polymerase and subsequently purified with RNeasy Mini Kits (Qiagen). An equimolar amount of reference cRNA was mixed with each experimental cRNA sample and was hybridized to Agilent Sureprint G3 Mouse GE 8x60K microarrays (Agilent) for 17 h at 65 °C in an Agilent SureHyb hybridization chamber. Immediately following the incubation period, the arrays were washed and scanned on an Agilent G2505B Scanner following the manufacturer’s recommended protocols. Feature Extraction 10.7.3.1 software (Agilent) was used to extract the gene expression data from the scanned images.

Normalization and analysis of the data were conducted in the R environment. The background fluorescence was measured using the negative control 3xSLv1 probes. Probes were flagged as absent (within the background signal) if the median signal intensities were less than the trimmed mean (trim = 5%) plus three trimmed standard deviations. Conversely, probes were considered present if at least four out of the five samples within a condition had signal intensities greater than three trimmed standard deviations above the trimmed mean of the 3xSLv1 probes. Data were normalized using Locally WEighted Scatterplot Smoothing (LOWESS) [33, 34], and outliers were identified using ratio intensity plots and heat maps of the raw and normalized data. Differentially expressed genes (DEGs)—increasing or decreasing relative to the lung tissue samples from age matched control mice—were determined using the MicroArray ANalysis Of VAriance (MAANOVA) library in R. This statistical model included the fixed effects slide and treatment condition, and was applied to the log2 of the relative intensities. The Fs statistic was used to test for treatment effects [35]. The *p* values for all statistical tests were estimated by the permutation method of that consisted of residual shuffling followed by the false discovery rate (FDR) approach to adjust for multiple comparisons [36]. A gene was considered significant if the FDR adjusted p value of the fold change in the experimental group relative to the control was less than 0.05.

### Pathway analysis of DEGs

Subsequent to normalization of the gene expression data, a short-list of DEGs was generated using the criteria of an absolute fold change ≥1.5 and an FDR *p*-value <0.05. Bar graphs summarizing the up and downregulated genes were generated with Prism 5 software (GraphPad Software, Inc., La Jolla, CA, USA). Lists of overlapping DEGs between experimental groups were generated with Venny [37] and proportional Venn diagrams were visualized with Venn Diagram Plotter (Pacific Northwest National Laboratory, Richland, WA, USA) (http://omics.pnl.gov/software/venn-diagram-plotter). DEGs associated with fibrosis were identified using the curated list in the Ingenuity Pathway Analysis knowledgebase (IPA, Ingenuity Systems, Redwood City, CA, USA).

### Real-time PCR (qPCR) validation of microarray data

Mouse fibrosis PCR arrays (PAMM-120Z, SABioscinces, Frederick, MD, USA) consisting of 86 genes were used to validate the microarray results. Approximately 800 ng of total RNA (*n* = 3 per group) from each of the experimental and control groups of STAT6 WT and KO mice was reverse transcribed using a RT2 first strand cDNA synthesis kit (SABiosciences, Frederick, MD, USA). qPCRs were conducted using RT2 SYBR Green qPCR Master Mix in a CFX96 Real-Time System (BioRad Laboratories, Mississauga, ON, Canada) according to the manufacturer’s instructions. Threshold cycle (Ct) values were normalized using *Gusb* as an internal control gene, and relative expression changes were each gene were determined using online PCR array data analysis software (SABiosciences, Frederick, MD, USA).

### Statistical analysis

Data are expressed as mean ± SEM. Statistical analysis was carried out using Prism Graphpad software. Student t-tests or two way ANOVAs were performed to assess the difference between MWCNT-treated and control groups, the statistical interaction of gene deficiency was then determined to assess the statistical significance relative to wild type. Differences with *p* < 0.05 were considered statistically significant.

## Results

### Neutrophilic inflammation in lungs induced by exposure to Mitsui-7 is mediated by IL-1 signaling

**Fig. 2:**
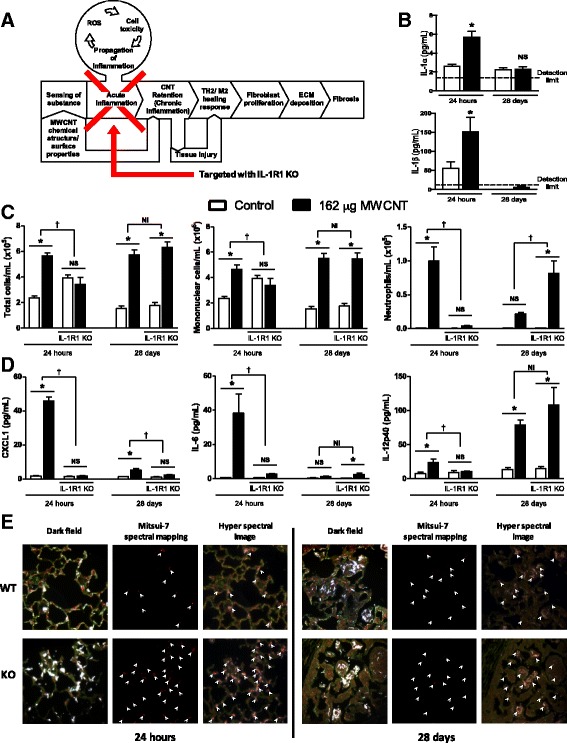
Acute inflammation in response to pulmonary MWCNT instillation is IL-1 dependent. C57BL/6 and IL-1R1 KO mice were intratracheally administered 162 μg of MWCNTs, and samples were collected 24 h and 28 days later. **a** Graphical representation of the key event targeted by IL-1R1 KO. **b** IL-1α and IL-1β were measured in BAL fluid of WT animals. The horizontal lines indicate limits of detection; IL-1 α – below 2.0 pg and IL-1 β – 0.04 pg. **c** The number of total cells, mononuclear cells, and neutrophils was determined from cytospin slides generated from BAL fluid and cell concentration measurements from BAL. **d** The inflammatory cytokines CXCL1, IL-6, and IL-12p40 were measured in BAL fluid. **e** Distribution of MWCNTs was determined by hyperspectral imaging in H&E stained histology samples. Data represent mean ± SEM. *n* = 4–5. Statistical analysis was performed using two-way ANOVA. *p < 0.05, NS = not significant, †statistical interaction with p < 0.05, and NI = no statistical interaction

Mitsui-7 has been shown to elicit a robust inflammatory response characterized by increased neutrophil influx and increased expression of inflammatory cytokines and chemokines acutely (within 24 h) after the exposure in experimental rodents [30, 38]. IL-1 mediated signaling has been shown to play a role in the acute inflammation induced by nanomaterials [39]. Acute inflammation involving chemokines and cytokine secretion has recently been identified as one of the key events in the MWCNT-induced AOP leading to lung fibrosis (Fig. 2a) [13]. To investigate the role of IL-1 in the acute inflammatory response elicited by MWCNTs, we first measured the two isoforms of IL-1, IL-1α and IL-1β, in the BAL obtained from WT and IL1-R1 KO mice intratracheally instilled with Mitsui-7 by ELISA. Mitsui-7 instillation significantly elevated the concentrations of IL-1α and IL-1β in the lungs of WT mice 24 h post-exposure. Both IL-1α and IL-1β levels reached detection limits at 28 days post-exposure (Fig. 2b) and the results were not significant.

Next, we conducted differential cell counts in BAL of WT and IL1-R1 KO mice 24 h and 28 days post-exposure to Mitsui-7 to quantify the inflammatory response. The results revealed significant differences between WT and IL-1R1 KO mice as determined by cellular infiltration into the lungs and the production of inflammatory cytokines. Significantly fewer cells were observed in the BAL of IL-1R1 KO mice compared to WT mice, mainly attributed to the complete abrogation of neutrophil influx in BAL fluid (Fig. 2c). However, this reduced cell number was not observed 28 days post exposure. Indeed, significantly more neutrophils were observed in IL-1R1 KO mice exposed to MWCNTs at this later time point. In alignment with lack of neutrophil influx, the levels of the IL-1 regulated inflammatory cytokines CXCL1, IL-6, and IL-12 remained at the basal levels following exposure to Mitsui-7 at 24 h (Fig. 2d) post-exposure. In comparison, the number of neutrophils and levels of inflammatory cytokines were significantly higher in Mitsui-7 treated WT mice compared to the matched vehicle only treated controls. The expression of CXCL1 was persistently attenuated at the 28 day timepoint in KO mice, but there was no suppression of IL-12 at 28 days (it was enhanced) post-exposure. This suggests that the suppression of some inflammatory mediators is transient and further confirms that IL-1 deficiency primarily affects the acute inflammatory response. The reduced expression of CXCL1 in the KO model is a particularly important observation as this is a key chemokine in the recruitment of neutrophils to the lung [40, 41]. These data indicate that IL1-R1 signaling is involved in Mitsui-7 induced inflammation and its deficiency disrupts the signaling by key inflammatory mediators resulting in an abrogated neutrophilic response; however, this lack of IL-1 signaling is compensated for by another mechanism at the later timepoint.

To understand if lack of neutrophil influx observed in IL-1R1 KO mice modifies clearance of these fibers from lungs, the dispersion of MWCNTs in the lungs of WT and IL-1R1 KO mice was assessed using the Cytoviva microscope, which combines darkfield enhanced imaging and hyperspectral profiling of the light scattered to identify MWCNTs. In H&E-stained lung sections of both WT and KO mice exposed to MWCNTs after 24 h, this qualitative analysis showed that the majority of MWCNTs appeared to be interacting with the epithelial cells that comprise the alveolar septa (Fig. 2e). To a lesser extent, MWCNTs could be viewed within phagocytic cells in the alveolar lumen, and the morphology of these cells identified them as macrophages. There was no difference in localization of MWCNTs in lung tissue of WT or KO mice; however, hyperspectral imaging suggested more MWCNT signal in the lungs of IL-1R1 KO mice compared to WT lungs at 24 h, but this could not be quantified. A large fraction of uniformly distributed MWCNTs was observed in lung sections of both WT and KO mice 28 days post-exposure. In the areas where MWCNTs were detected, the alveolar septa were thick (Fig. 2e) and MWCNTs seemed to be located in the midst of these thickened areas of epithelial structure. No differences between WT and KO samples were apparent.

### Deficiency in IL-1 signaling does not affect the pathogenesis of MWCNT-induced fibrotic disease

**Fig. 3:**
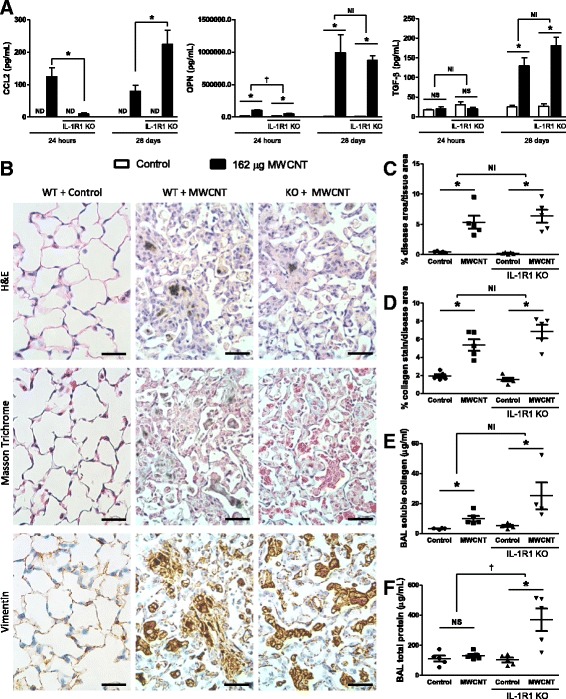
Fibrotic disease is not attenuated 28 days after MWCNT exposure. C57BL/6 and IL-1R1 KO mice were intratracheally administered 162μg of MWCNTs, and samples were collected 24 h and 28 days later. **a** The pro-fibrotic genes CCL2, osteopontin (OPN), and total TGF-β levels including the active form were measured by ELISA. **b** Representative histology images of the diseased area of lungs 28 days after MWCNT instillation compare WT and IL-1R1 KO mice. These images were obtained from slides stained with H&E, Masson Trichrome for collagen deposition (blue), and immune-staining for Vimentin, a surface marker of fibroblasts (brown). The scale bar represents 50 μm. **c** Entire longitudinal cross sections of the lungs were imaged and the disease area verses the total lung area were determined to quantify the pathology in H&E stained samples 28 days post exposure. **d** Representative images of pathology were taken of the Masson trichrome stained slides and the amount of collagen positive stain was quantified and normalized as a percent of area imaged. Non-diseased areas from exposed mice were used as controls. **e** Soluble collagen was measured in the BAL fluid at the 28 day time point, and **f** total protein was measured in BAL as well. Data represent mean ± SEM. n = 4–5. Statistical analysis was performed using two-way ANOVA. *p < 0.05, NS = not significant, †statistical interaction with p < 0.05, and NI = no statistical interaction

Next, we sought to assess fibrotic disease development in IL-1R1 KO mice. First, we measured prototypical fibrotic mediators in the BAL fluid of MWCNT-exposed mice. The products of the pro-fibrotic genes CCL2, osteopontin (OPN), and TGF-β were all increased following MWCNT exposure in WT mice, though TGF-β was only increased at the 28-day time point (Fig. 3a). In comparison, the pro-fibrotic chemokine CCL2 was decreased in KO mice 24 h after MWCNT exposure, but increased in KO mice 28 days post-exposure. The upregulation of OPN was dampened in KO mice 24 h post exposure, with no significant difference between WT and KO mice at the 28-day time point. No difference was observed in the induction of TGF-β between WT and KO mice. These data suggest a role for IL-1 signaling in the initial induction of some pro-fibrotic mediators; however, the role of IL-1 signaling is ultimately redundant as these pro-fibrotic mediators can be induced in the absence of IL-1R1 at a later time point.

We assessed the morphological changes to lung tissue 28 days post-MWCNT administration (Fig. 3b). The H&E stain (Fig. 3b upper panel) was used to identify areas of thickened alveolar septa. H&E stained histology samples were prepared and the entire longitudinal cross section of both lungs were imaged. The total area of the lungs and diseased areas, as identified by thickened alveolar septa, were determined (procedure outlined in Additional file 1: Figure S1). MWCNTs led to a significant increase in quantifiable disease area compared to vehicle treated matched control lungs, with no difference observed between WT and KO mice (Fig. 3c).

Masson trichrome stain (Fig. 3b middle panel) was used to assess whether the exposures led to increased deposition of collagen, which is indicative of fibrosis in the metaplastic lesions. Increased collagen deposition was interspersed throughout the observed lesions in both WT and KO mice, consistent with previous observations in MWCNT-exposed lungs [30]. Collagen positive stain was quantified in Masson trichrome stained samples and showed a significant increase in collagen deposition induced by MWCNTs, but with no differences between WT and IL-1R1 KO mice (Fig. 3d). Additionally, soluble collagen was measured in BAL fluid. Significantly more collagen was measured in the BAL fluid of MWCNT-exposed WT mice as well as in MWCNT-exposed IL-1R1 KO mice (Fig. 3e).

Immunohistochemistry analysis for the fibroblast marker vimentin was undertaken to further characterize the lesions (Fig. 3b lower panel). The lesions in MWCNT-instilled lungs displayed increased vimentin signal indicating increased fibroblast proliferation in these tissues, which is consistent with pulmonary fibrosis. No differences were apparent in the structure of the diseased tissue between WT and IL-1R1 KO mice.

In addition to the fibrotic changes, we assessed proteinosis by measuring the total protein content of BAL fluid. IL-1R1 deficiency resulted in increased accumulation of protein in the lungs over the WT mice, suggesting that IL-1R1 KO negatively affects the ability of the lungs to deal with MWCNT exposure.

Collectively, these data suggest that although IL-1 signaling deficiency attenuates the acute inflammatory response elicited by MWCNTs, there is no subsequent difference in the development of fibrotic lesions in WT and IL-1R1 KO mice.

### IL-1R1 deficiency initially impacts over half of all MWCNT-induced differentially expressed genes and fibrotic genes

**Fig. 4:**
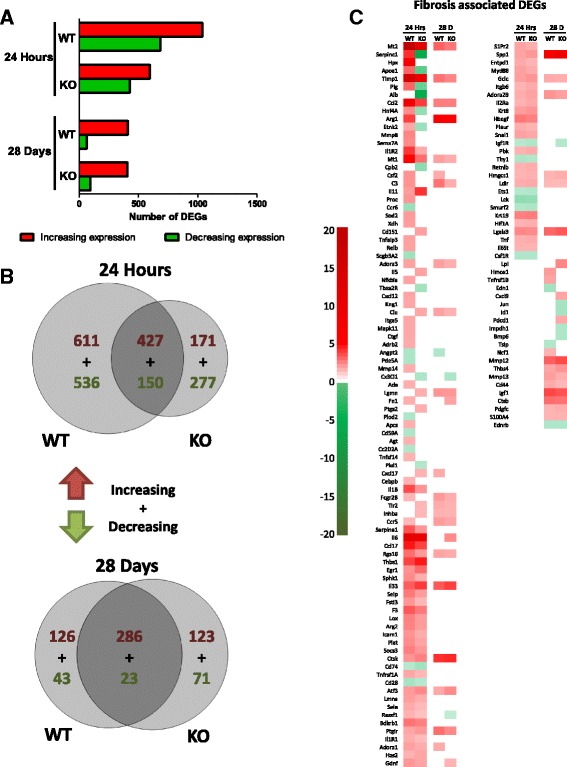
IL-1 deficiency differentially affects more MWCNT-induced fibrotic genes at 24 h compared to 28 days. RNA was isolated from the lung tissue of MWCNT-administered mice 24 h and 28 days post-exposure. **a** The number of DEGs is visualized by a bar chart and the number of significant (at least a 1.5 fold change and an FDR adjusted p < 0.05) DEGs between WT and IL-1R1 KO mice is indicated for both time points. **b** Venn analysis was used to visualize the degree of overlap between increasing and decreasing DEGs at the 24 h and 28 day time points. **c** A heat map visualizing all of the DEGs involved in inflammation and fibrosis is shown. The genes are ordered based on the difference in expression between WT and IL-1R1 KO mice

Whole-transcriptome analysis was utilized to more understand the effects of IL-1R1 deficiency on the pulmonary response to MWCNT exposure. Total RNA was isolated from the lung tissue of mice 24 h and 28 days after exposure to MWCNTs and global gene expression changes were assessed by microarrays in both WT and KO mice. This analysis identified 1724 DEGs in WT mice, with 1038 upregulated genes and 686 downregulated genes 24 h after MWCNT exposure (Fig. 4a). Fewer DEGs were identified in the IL-1R1 KO mice at the 24-h time point, with 1025 DEGs observed, 598 of which were upregulated and 427 DEGs were downregulated. The biological response to MWCNTs seemed less pronounced 28 days after exposure as 478 DEGs were observed in WT mice (412 upregulated and 66 downregulated). Similar results were observed in the KO mice at 28 days with 503 DEG identified (409 upregulated and 94 downregulated).

Venn diagrams were used to assess the similarities and differences in DEGs between WT and KO mice (Fig. 4b). KO mice exhibited fewer DEGs at the 24 h post-exposure time point relative to WT mice. Moreover, a large subset of genes was only differentially expressed in KO mice, and over half of the DEGs found in WT mice were unique to WT at 24 h time-point. At 28 days post exposure, about a third of the DEGs in WT mice were not differentially expressed in KO mice, with an almost equal number of DEGs only expressed in the KO. The results suggest that initial response to MWCNTs is attenuated by the absence of IL-1 signaling; however, this attenuation is transient as by 28 days post-exposure there is a much lesser degree of difference between the WT and KO mice.

The IPA knowledgebase was used to identify DEGs from the microarray experiments that are associated with fibrosis. The results are visualized by a heat map (Fig. 4c). The heat map is ordered by the greatest difference in expression between WT and KO mice. Genes such as *Mt2*, *Serpinc1*, *Hpx*, and *APoa1* were induced by MWCNT exposure, and attenuated in KO mice; however, this attenuation was less pronounced or undetectable at the 28 day time point. Generally, the difference in expression of genes associated with fibrosis between WT and KO mice 28 days after exposure was modest, which is consistent with the inability to detect a difference in the development of fibrotic disease at the 28 day time point.

### STAT6 Signaling contributes to neutrophilic inflammation in lungs induced by exposure to Mitsui-7

**Fig. 5:**
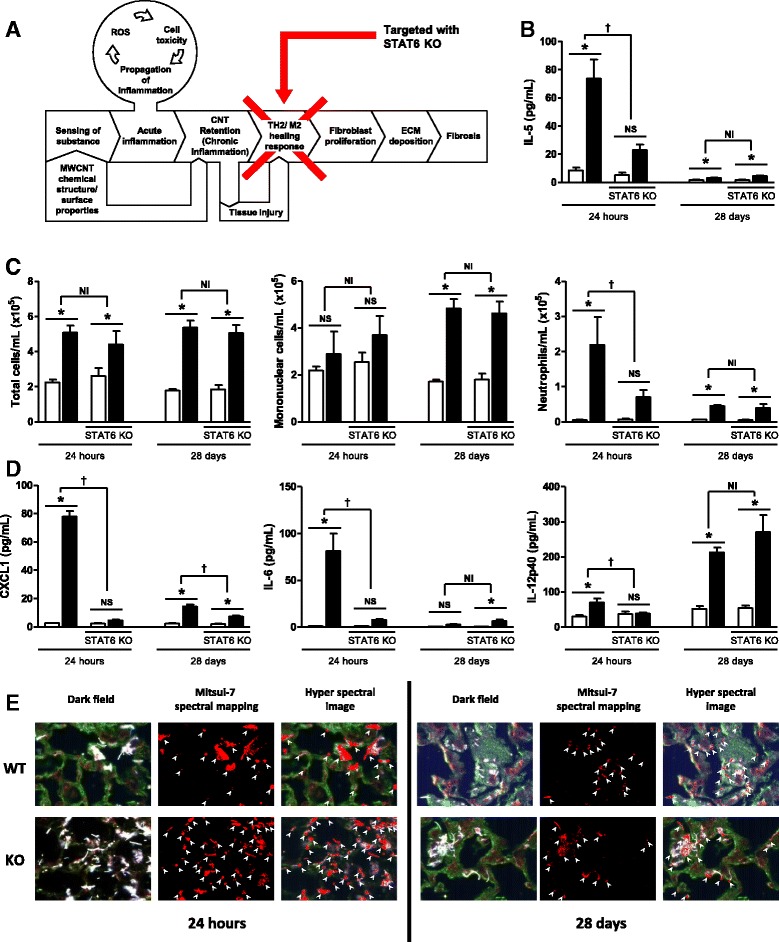
Acute inflammation in response to pulmonary MWCNT instillation is partially STAT6 dependent. C57BL/6 and STAT6 KO mice were intratracheally administered 162 μg of MWCNTs, and samples were collected 24 h and 28 days later. **a** Graphical representation of the key event targeted by STAT6 KO. **b** IL-1α and IL-1β were measured in BAL fluid of WT animals. **c** The number of total cells, mononuclear cells, and neutrophils was determined from cytospin slides generated from BAL fluid and cell concentration measurements from BAL. **d** The inflammatory cytokines CXCL1, IL-6, and IL-12p40 were measured in BAL fluid. **e** Distribution of MWCNTs was determined by hyperspectral imaging in H&E stained histology samples. Data represent mean ± SEM. n = 4–5. Statistical analysis was performed using two-way ANOVA. *p < 0.05, NS = not significant, †statistical interaction with *p* < 0.05, and NI = no statistical interaction

STAT6 mediated signaling plays an integral role in the development of fibrosis in animal models [24, 25]. STAT6 has also been shown to be part of the Th2 response activated by MWCNT exposure [26], and this healing response is a key event in the MWCNT-induced adverse outcome pathway leading to lung fibrosis (Fig. 5a) [13]. Following the same experimental protocol as the IL-1R1 KO experiments, inflammation and lung pathology 24 h and 28 days post MWCNT exposure in STAT6 WT and KO mice was investigated. First, the importance of STAT6 in the production of Th2-associated genes was confirmed by measuring IL-5 in the BAL of MWCNT-exposed mice. As shown in Fig. 5b, MWCNT instillation significantly elevated the concentrations of IL-5 in the lungs of STAT6 WT mice, but this response was significantly attenuated in STAT6 KO mice. This reduction was not observed at the 28 day time point, but IL-5 was induced at much lower levels in STAT6 WT and KO mice at this time point.

Next, lung inflammation was assessed by conducting differential cell counts in the BAL of WT and STAT6 KO mice at 24 h and 28 days post-exposure to Mitsui-7 (Fig. 5c). No difference was observed at either time point with the total cell count or mononuclear cell counts in STAT6 KO mice 24 h post exposure; however, significantly less neutrophils were observed in the KO mice. With respect to inflammatory cytokines, STAT6 KO mice produced significantly less CXCL1, IL-6, and IL-12, as measured in BALF by ELISA at 24 h post Mitsui-7 exposure (Fig. 5d). Indeed, there was no significant increase above control levels at 24 h-post exposure for any of these cytokine. CXCL1 continued to be significantly suppressed at the 28 day time point, while IL-6 and IL-12 responses were similar in both WT and KO mice at this later time point. These data demonstrate that STAT6 signaling contributes to the acute inflammatory response to MWCNTs, but to a lesser extent than IL-1R1 signaling.

To assess the distribution of fibers in the lungs of STAT6 KO mice, the dispersion of MWCNTs was visualized using the Cytoviva microscope at 24 h and 28 days post-exposure. This qualitative analysis showed that the majority of MWCNTs appeared to be interacting with the epithelial cells that comprise alveolar septa, with some present in phagocytic cells of the lung lumen (Fig. 5e). These observations were similar to those made in IL-1R1 KO mice and their corresponding controls. Also, similar to IL-1R1 KO mice, the lungs of STAT6 KO mice appeared to contain a higher burden of MWCNTs, but could not be quantified. This observation would be consistent with inflammation playing an important clearance role early after MWCNT administration.

### Deficiency in STAT6 signaling reduces MWCNT-induced fibrotic disease

**Fig. 6:**
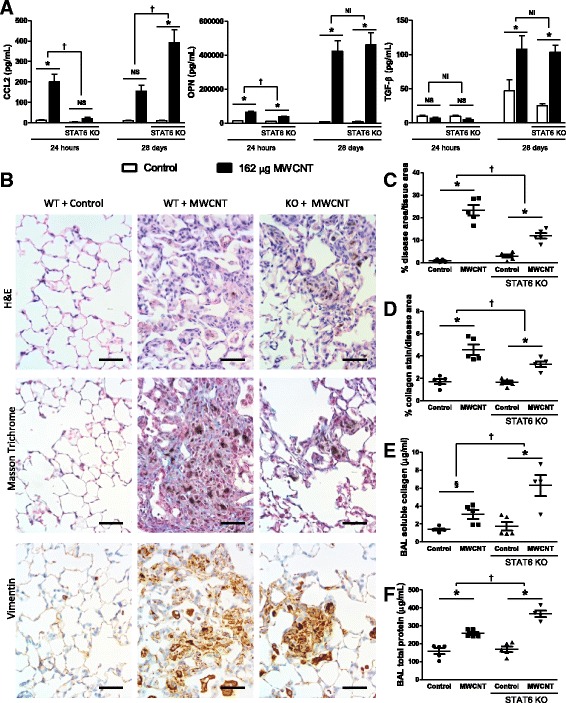
Fibrotic disease but not traditional fibrotic markers are attenuated 28 days after MWCNT exposure. C57BL/6 and STAT6 KO mice were intratracheally administered 162 μg of MWCNTs, and samples were collected 24 h and 28 days later. **a** The pro-fibrotic genes CCL2, osteopontin (OPN), and total TGF-β levels including the active form were measured by ELISA. **b** Representative histology images of the diseased area of lungs 28 days after MWCNT instillation compare WT and STAT6 KO mice. These images were obtained from slides stained with H&E, Masson Trichrome for collagen deposition (blue), and immune-staining for Vimentin, a surface marker of fibroblasts (brown). The scale bar represents 50 μm. **c** Entire longitudinal cross sections of the lungs were imaged and the disease area versus the total lung area was determined to quantify the pathology in H&E stained samples 28 days post exposure. **d** Representative images of pathology were taken of the Masson trichrome stained slides, and the amount of collagen positive stain was quantified and normalized as a percent of area imaged. Non-diseased areas from exposed mice were imaged as controls. **e** Soluble collagen was measured in the BAL fluid at the 28 day time point, and **f** total protein was measured in BAL as well. Data represent mean ± SEM. n = 4–5. Statistical analysis was performed using two-way ANOVA. *p < 0.05, NS = not significant, †statistical interaction with *p* < 0.05, and NI = no statistical interaction

Similar to the IL-1R1 KO studies, the expression of prototypical mediators of fibrotic disease were investigated. The products of the pro-fibrotic genes CCL2, osteopontin (OPN), and TGF-β were measured in the BAL of MWCNT-exposed mice. In concordance with our previous findings, all of these genes were elevated following MWCNT exposure in WT mice, with the exception of TGF-β at the 24 h time point (Fig. 6a). CCL2 response was diminished relative to wild type (not above its matched controls) in KO mice 24 h after MWCNT exposure. However, CCL2 was not only significantly greater than controls in KO mice 28 days post exposure, but was also a significantly greater response than exposed WT at this time point. OPN was also dampened in KO mice relative to WT 24 h post exposure, but no significant difference was found between WT and KO mice at the 28-day time point. No difference was observed in the induction of TGF-β between WT and KO mice. These data imply that STAT6 signaling plays an important role in the early expression of these pro-fibrotic mediators, but that STAT6 signaling is not critical for the upregulation of these mediators 28 days post Mistui-7 exposure.

Morphological changes were assessed in lung tissue 28 days after MWCNT administration (Fig. 3b). In order to quantify the fibrotic area in lungs, the entire longitudinal cross section of both lungs was imaged from each H&E stained histology samples. Representative images of the diseased area suggest that MWCNTs caused a significant increase in quantifiable disease area in WT mice, and that the thickening of the alveolar septa was reduced in STAT6 KO mice (Fig. 6c). Collagen positive stain was quantified in Masson trichrome stained samples and showed a significant increase in collagen deposition induced by MWCNTs in WT mice. However, significantly less collagen positive stain was quantified in STAT6 KO mice (Fig. 3d) relative to the WT response. Additionally, soluble collagen was measured in BAL fluid. Significantly more collagen was found in BAL fluid from MWCNT-exposed WT as well as STAT6 KO mice (Fig. 6e) but a larger effect was observed in the KO mice. Immunohistochemistry for the fibroblast marker found that the lesions in STAT6 KO mice stained positive for vimentin similarly to WT mice. The images support the involvement of STAT6 signaling in the development of MWCNT-induced lung fibrosis.

Additionally, we assessed proteinosis by measuring the total protein content of BAL fluid in these experiments and, similar to IL-1R1 KO mice, STAT6 deficiency lead to an increased amount of protein accumulating in the lungs (Fig. 6f).

### STAT6 Deficiency initially impacts half of all MWCNT-induced differentially expressed genes and leads to sustained attenuation of expression of specific fibrotic genes

**Fig. 7:**
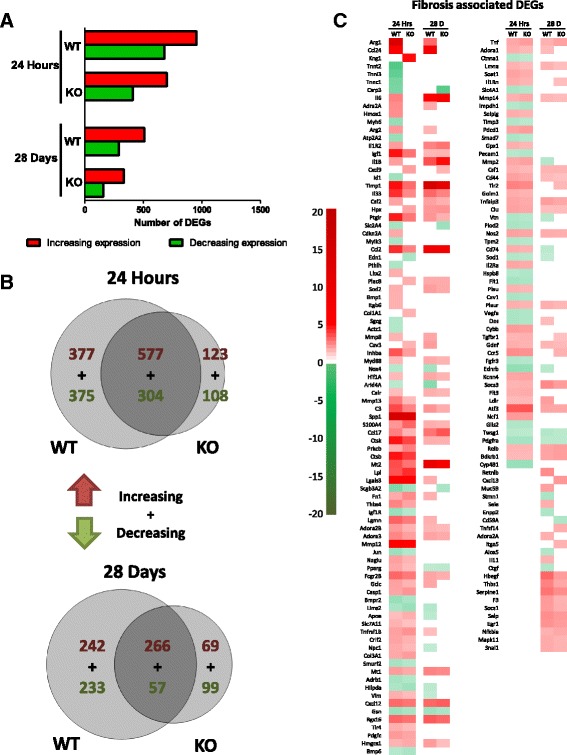
STAT6 deficiency suppresses fibrotic genes 24 h and 28 days post MWCNT exposure. RNA was isolated from the lung tissue of MWCNT-administered mice 24 h and 28 days post-exposure. **a** The number of differentially expressed genes (DEGs) is visualized by a bar chart and the number of significant (at least a 1.5 fold change and an FDR adjusted p < 0.05) DEGs between WT and STAT6 KO mice is indicated for both time points. **b** Venn analysis was used to visualize the degree of overlap between increasing and decreasing DEGs at the 24 h and 28 day time points. **c** A heat map visualizing all of the DEGs involved in inflammation and fibrosis is shown. The genes are ordered based on the difference in expression between WT and STAT6 KO mice

The effects of STAT6 deficiency on the pulmonary response to MWCNT exposure was assessed by whole transcriptome analysis 24 h and 28 days after exposure to MWCNTs. This analysis revealed 1633 DEGs in WT mice, with 954 upregulated genes and 679 downregulated genes 24 h after MWCNT exposure (Fig. 7a). Fewer DEGs were found in the KO mice at the 24-h time point, with 1112 DEGs observed, 700 of which were upregulated and 412 of which were downregulated. The biological response to MWCNTs was less pronounced 28 days post exposure, with 798 DEGs in WT mice (508 upregulated and 290 downregulated). Similarly, STAT6 KO mice at 28 days had reduced response relative to 24hs, with 503 DEGs (335 upregulated and 156 downregulated).

To assess the similarities and differences in the gene expression profiles of WT and KO mice, Venn diagrams were produced (Fig. 7b). This analysis visualized the effect STAT6 deficiency has on the biological response to MWCNT. About 75% of the DEGs in STAT6 KO mice were shared with the WT mice at the 24 h time point. Thus, a relatively small proportion of unique DEGs were found in STAT6 KO mice. This suggests that STAT6 deficiency suppresses a portion of the response to MWCNT rather than altering the response to a different set of DEGs. This pattern of gene expression was found for both the 24 h and 28 day time points, indicating that the effects of STAT6 deficiency affect both the acute and chronic response to MWCNTs in a similar capacity.

We focused our subsequent analysis on genes associated with the development of fibrosis as above. A heat map of all the DEGs associated with the fibrosis pathway showed a subset of DEGs whose expression differs between wild type and KO mice, and a subset of genes that were largely unaffected by STAT6 deficiency. The heat map is ordered by the greatest difference in expression between WT and KO mice. These data indicate that the fibrotic genes *Arg1*, *Arg2*, *Ccl24*, and *Adra2A* were significantly upregulated in WT mice, but not in KO mice, at both times points post MWCNT exposure. Of note, *Arg1*, *Arg2*, *Ccl24*, and *Retnlb* were significantly altered in WT mice at 28 days but not in STAT6 KO mice. These genes are positively associated with alternatively activated M2 macrophages [42, 43]. M2 macrophages are crucial contributors to the development of fibrosis [44], and these data implicate M2 macrophage-associated genes with the development of MWCNT-induced fibrosis (Fig. 7c).

**Fig. 8:**
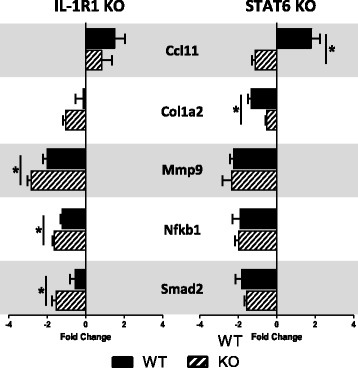
PCR array identified the suppression of different fibrotic genes in IL-1R1 KO and STAT6 KO mice. A PCR array of fibrosis-associated genes was performed on RNA isolated from C57BL/6, IL-1R1 KO, and STAT6 KO mice 28 days after MWCNT challenge. Five genes were identified as significantly different between KO and WT mice exposed to MWCNTs in either STAT6 or IL-1R1 KO mice. Data represent mean ± SEM. *n* = 3. Statistical analysis was performed using t-tests. *p < 0.05

To further search for an explanation for the differences in fibrosis development between IL-1R1 and STAT6 KO mice, the expression of 86 individual genes that are suggested to be associated with fibrosis was assessed in lung tissues derived from both the IL1-R1 and STAT6 experiments by RT-PCR (Fig. 8). This revealed 5 specific genes whose expression was significantly different in the STAT6 KO model 28 days post MWCNT exposure compared to the MWCNT-treated STAT6 WT mice: *Ccl11*, *Col1a2*, *Mmp9*, *Nfkb1*, and *Smad2*. Of these, only *Ccl11* was upregulated by MWCNT exposure, and this expression was significantly attenuated in STAT6 KO mice. *Ccl11* expression was not significantly altered in IL-1R1 KO mice. These data suggest that in addition to M2 macrophage-associated genes mentioned above, *Ccl11* is another gene that differentiates the response between STAT6 KO and IL-1R1 KO mice.

## Discussion

An effective human health risk assessment strategy to identify the potential pathological effects of NMs, including MWCNTs, will require a detailed understanding of the biological mechanisms involved. It has been well documented that exposure of experimental rodents to MWCNTs results in chronic inflammation and lung fibrosis [26, 28, 30]. It has also been suggested that lung inflammation induced both acutely and at the adaptive phases following exposure to MWCNTs plays an important role in the fibrotic disease process [13]. However, no studies have been conducted to systematically investigate the role of inflammation in MWCNT-induced fibrosis.

In this study, knock out models were used to specifically inhibit the acute and chronic phases of inflammation, as described in the AOP, to define the essentiality of two inflammatory pathways to the overall pathogenesis of lung fibrosis induced by MWCNTs [13]. Specifically, the IL-1 signaling pathway was targeted to inhibit the acute inflammatory event, and STAT6 mediated signaling was targeted to inhibit the healing response associated with the chronic inflammatory phase. Mitsui-7 is known to induce fibrotic lesions in rodent models, and the dose and post-exposure timepoints in this experiment were chosen to: (1) keep the experimental parameters consistent with previous studies [30]; (2) allow for more meaningful comparative analyses in the future; (3) be relevant to occupational exposure levels. Although the dose used in this study is high, it enabled greater sensitivity in measuring the potential differences in the response to MWCNTs between WT and KO mice. The study results showed that both IL-1R1 and STAT6 mediated signaling are involved in acute inflammation induced by MWCNT exposure. The results also showed that while IL1-R1 signaling is redundant for lung fibrosis, STAT6 mediated Th2 response is essential in the process of fibrosis induced by biopersistent MWCNTs.

The role of IL-1 signaling in fiber-induced inflammation and fibrosis has not been investigated. Although it has been established that disrupting inflammatory mediators attenuates fibrosis with other causative agents [11], these data have been generated in experimental models where the substances used to induce fibrosis exhibit different physical and chemical properties from high aspect ratio materials such as MWCNTs. To our knowledge, no published study has directly targeted IL-1R1 in a model of asbestos exposure. It has been shown that mice deficient in the inflammasome component NALP3—a necessary component for IL-1β processing and release—mount an attenuated inflammatory response when exposed to asbestos [45]; however, its implication to asbestos-induced lung pathology has not been investigated. Rydman et al. targeted IL-1 signaling in a model of MWCNT exposure [21], and showed that IL-1R1 deficiency attenuates acute inflammation, which is consistent with the results of the present study. Interestingly, Rydmen et al. did not observe reversal of the attenuated inflammatory process at 28 days post-exposure, which is in contrast to the results observed here. This discrepancy could be due to the lower doses used by Rydmen et al., which may have led to effective clearance of MWCNTs by other mechanisms. However, the study did not assess the impacts of the attenuated inflammation on fibrotic process. In Gristman et al., the role of IL-1 signaling in MWCNT-induced fibrotic pathology was evaluated [46]. This study showed that acute neutrophilic response following exposure to MWCNTs was dampened at 24 h post-exposure but the fibrotic pathology was exacerbated in MWCNT-treated IL-1R1 deficient mice at 28 days post-exposure compared to the wild type mice, suggesting that lack of neutrophilic clearance of MWCNTs acutely after the exposure may have exacerbated the disease response in these mice [46]. In support of this hypothesis, darkfield microscopy revealed higher amount of MWCNTs in lungs of IL-1R1 deficient mice at 24 h post-exposure compared to the wild type mice in the present study. Moreover, statistically significant increases in TGF-β protein levels, a potent pro-fibrotic cytokine and a strong immunosuppressive molecule, at the acute time point (24 h) after exposure to MWCNTs. In addition, moderate (non-significant) increases in the total collagen deposition was also observed in MWCNT-treated IL-1R1 deficient mice. We further expand on the findings of these studies mentioned above, and show that MWCNT-induced lung fibrosis proceeds in the absence of acute phase IL-1 signaling mediated inflammation and demonstrate that it may be partially driven by STAT-6 signalling. Few other studies have shown disengagement between innate immune responses and ultimate lung fibrosis in a mouse model after exposure to silica [47]*.* This study characterized the role of innate immune responses in lung fibrosis using 11 individual knock out mouse models lacking different members of IL-1 family including, ASC, NALP3, IL-1R, IL-18R, ILK-33R, IL-1a and IL-1b, as well as other innate immune response mediators such as MyD88, TLR2/4, TLR3, TRIF, IL-23p19, and TCRδ. The study concluded that fibrosis induced by silica can occur in the absence of innate inflammatory responses [47]. In another study, inhibition of innate immune responses via treatment with dexamethasone, COX inhibitor piroxicam or the phosphodiesterase 5 inhibitor sildenafali was shown to have no impact on total collagen content in mouse lungs exposed to silica [48]. Although silica used in Lo Re et al. differs in its properties compared to the MWCNT type used in the present study, the results are supportive of the findings.

Our results are unique from the well-established bleomycin model of fibrosis, in which the end results of tissue injury, chronic inflammation, and pulmonary fibrosis induced by bleomycin were attenuated in IL-1R1 deficient mice [20, 49]. While the gene expression patterns following exposure to bleomycin and MWCNTs show some similarities, the apparent differences in the end toxicity outcome in IL-1R1 KO mice can be attributed to the nature of the fibrosis-inducing substance. Bleomycin is a small molecule drug with a half-life of less than an hour in the lungs of C57BL/6 mice [50], while the hyperspectral imaging of mouse lungs exposed to Mitsui-7 in the present study indicates that the fibers are not readily cleared from the lungs and persist for at least 28 days post exposure. Although the acute response to both bleomycin and MWCNTs may be IL-1 dependent, the biopersistant nature of MWCNTs resulting in chronic interaction with the pulmonary environment may oblige other inflammatory mechanisms compensating for the IL-1R1 deficiency, explaining the observed reengaged neutrophil influx at 28 days post-exposure. It is also possible that the late inflammatory and fibrotic responses are not dependent on the IL-1 axis, including in WT mice. Interestingly, in STAT6 KO mice, expression of IL-1α was suppressed at 24 h post-exposure (data not shown). These results imply that it is a possibility that suppression or abrogation of lung fibrosis may require parallel inactivation of both acute and adaptive immune responses. Further studies involving both dose and time series are needed to fully elucidate the role of IL-1 axis in MWCNT-induced lung pathology. Considering the critical role of the inflammatory response in the maintenance of cellular homeostasis, the results presented suggest that it may not be possible to achieve complete and sustained abrogation of inflammation by targeting a single gene or pathway in the presence of a persistent active toxicant. It can also be suggested that abrogation of innate immune responses acutely after the exposure, may trigger activation of immunosuppressive Foxp3+ regulatory T (T reg) cells that exhibit profibrotic acitvities and secrete high levels of TGF-b. Persistence and accumulation of immunosuppressive T reg cells has been shown to contribute to silica induced lung fibrosis in mice by TGF-b autocrine signaling pathway mediated secretion of platelet-derived growth factor resulting in stimulation of fibroblasts [51].

Collectively, our findings suggest that the early biological response is extensively altered by IL-1 deficiency, and although IL-1α and IL-1β may not be crucial components of MWCNT-induced fibrosis, this observation hints at an important role for these genes in sensing the damage elicited by MWCNTs. The sensing of damage is a key initiating step in the propagation of an inflammatory response, and this is accomplished by recognizing damage associated molecular patterns (DAMPs) [52]. IL-1 family members have been proposed as canonical DAMPs that initiate inflammatory responses after tissue damage [53]. This hypothesis is supported by experiments that target IL-1α to ameliorate the inflammation induced by necrotic cells [54]. MWCNTs are cytotoxic to certain cell lines [55], and it is likely that the specific properties of pathogenic MWCNTs allow for interactions with pulmonary cells that results in necrosis. The DAMPs that are released by these necrotic events then initiate the inflammatory response associated with MWCNT inhalation. IL-1α and IL-1β could then be considered important DAMPs or early mediators in initiating MWCNT-induced inflammation. Adopting this understanding of the role of IL-1 signaling in the response to MWCNT inhalation explains why IL-1R1 is redundant at chronic time points in our model. Many molecules can act as DAMPs, and it is believed that DAMP inflammatory signaling is fundamental for survival, which may necessitate redundant signaling pathways consistent with our observations [56]. Considering IL-1α as a DAMP and and IL-1β as one of the sensitive early responder implies that while these genes may not be crucial mediators of MWCNT-induced fibrosis, they may be considered important biomarkers of the MWCNT-induced lung damage that ultimately leads to fibrosis. As such, IL-1α and IL-1β may still be useful in future MWCNT screening strategies.

One of the other long term responses to MWCNTs in IL-1R1 KO mice was the development of pulmonary alveolar proteinosis. While its relevance to the end pathology of MWCNT-driven lung fibrosis in mice is not known at present, similar findings of increased BALF protein was also reported by Huaux et al. in IL-1R1, IL-1α or Myd88 deficient mice but not in IL-1β or ASC deficient mice exposed to silica particles [57]. The authors suggested that proteinosis was associated with limited clearance of particles and that in the absence of IL-1R1 or IL-1α, particle clearance is greatly impacted. In the present study, suppression of acute inflammatory responses and BALF proteinosis was also observed in STAT6 KO mice, which showed suppressed IL-1α expression at 24 h post-exposure (unpublished data) and acute inflammatory responses, suggesting that indeed, suppression of IL-1α axis or acute innate immune responses in general, resulting in impaired clearance of particles may be causal to proteinosis.

The presence of toxic fibers, their physical interaction with surrounding tissue, and resulting tissue injury subsequently induce a Th2 response leading to regulation of inflammation, which seems to play a detrimental role in MWCNT-induced lung fibrosis [58, 59]. Targeting of Th2-mediated signaling by STAT6 KO clearly showed the criticality of this signaling in the development of fibrosis. The Th2 response is central to allergic disease, and our findings are consistent with other studies that have shown a relationship between MWCNT exposure and allergic disease. Experimental models of allergic airway disease are exacerbated by MWCNT exposure [60], and MWCNTs can be used as an adjuvant to sensitize mice to an allergen [61]. In a study by Katwa et al. it was shown that activation of Th2 response involving IL-1 like cytokine IL-33 is critical for acute inflammation and lung fibrosis induced by carbon based nanomaterials including MWCNTs [62]. STAT6 has been shown to be activated by MWCNT exposure, and our study represents the first evidence of the significant role it plays in MWCNT-induced fibrosis [26]. Of note, STAT1 has been investigated in a model of MWCNT and allergen exposure [63], and found to play an opposite role to our findings with STAT6.

Our data suggests the role of STAT6 signaling in our model may be specifically tied to the role of M2 macrophages. The term M2 macrophage typically describes an alternatively activated macrophage that can fill a spectrum of roles from immune regulation to wound healing (reviewed in [64]). Enhancing the activation of M2 macrophages has been shown to accelerate the healing process [65], and the ability of M2 macrophages to facilitate healing implicates excessive and prolonged M2 activation as a mechanism of fibrosis development. Bleomycin-induced pulmonary fibrosis models indicate a critical role for M2 macrophages in disease pathogenesis [66, 67]. However, the role of M2 macrophages in MWCNT-induced pathology has been largely unstudied, although M2 macrophage markers have been observed after MWCNT stimulation in a culture system [68]. Our data expand on this in vitro observation, and suggest that the ability of MWCNTs to lead to sustained activation and presence of M2 macrophages within lung tissue is a KE in the development of MWCNT-induced pathology. Specifically, the M2 macrophage-associated genes *Arg1*, *Arg2*, *Ccl24*, and *Retnlb* could be incorporated into future screening strategies as disease biomarkers. However, it is important to note that the present study did not specifically assess M2 macrophages and thus, further studies are needed to support these conclusions.

The redundancy of IL-1 signaling in the development of fibrosis highlights the strength of applying an AOP framework to guide mechanistic studies. Inflammation occurs early in the response to inhaled MWCNTs and is such a fundamental response to so many insults that multiple pathways are available to eventually compensate for the lack of an initial inflammatory response. The AOP framework readily enables the identification of downstream events for research that may be more specific to the AO, and thus directs more meaningful mechanistic studies. In our experiment this led us to target wound healing as a later KE. Our work here led to an unexpected finding on the effect of STAT6 deficiency on the acute inflammatory response. STAT6 is traditionally known as a signaling pathway that is initiated later in the response to inflammatory stimuli and is associated with the eventual suppression of inflammation. However, STAT6 KO mice elicit a blunted neutrophilic response after infection with pneumocystis [69]. Our experiment provides additional insight into the role of STAT6 in the acute inflammatory response, as well as the subsequent Th2 response. Ultimately, placing our mechanistic data within the context of an AOP helps place our negative results related to IL-1R1 deficiency into perspective and strengthens the observed role of STAT6 in MWCNT-induced pathology. Overall, the AOP framework facilitates the identification of data gaps, uncertainties and inconsistencies in hypothetical pathways. The weight of evidence approach applied in AOP development is also a very useful tool enabling rapid identification of necessary hypothesis-directed research.

In the present study, a potential link between lung inflammation and lung fibrosis was explored with the hypothesis that inflammation is essential to the process of fibrosis and that it precedes and accompanies fibrosis. The results presented show that some components of the inflammatory process may be involved in the fibrotic disease development but that the hypotheses cannot be generalized to all types of inflammatory processes. As reviewed in Luzina et al. the interaction between inflammation and fibrosis could be 1) direct – the underlying mechanisms that drive MWCNT-induced inflammation also drive the fibrosis pathology, 2) indirect – the mechanisms driving MWCNT-induced inflammation and lung fibrosis are initiated independently but some level of interaction between the two processes occur and may contribute to the disease progression, and 3) independent or no interaction – inflammation and fibrosis occur independent of each other [70]. From the present study, it can be concluded that the relationship is both direct and indirect, depending on the type of inflammatory processes (specific pathways or molecules) and inflammatory phases (innate versus adaptive or resolving phase) considered. Moreover, for nanomaterials, this interaction or the extent of this interaction could be dictated by their properties and exposure duration. Thus, despite the observation that IL-1R1 deficiency did not alter the final fibrotic outcome following exposure to MWCNTs, its involvement in the disease process cannot be entirely ruled out. Moreover, the present study only considered one acute and one sub-chronic post-exposure time point. A study including a range of post-exposure time points may be necessary to fully appreciate the involvement of the acute inflammatory phase in the development of MWCNT-induced lung fibrosis.

While the present study has specifically focused on the potential link between MWCNT-induced lung inflammation and lung fibrosis, other studies have proposed that fibroblasts proliferation and collagen synthesis to be the critical events that drive the CNT-induced lung fibrosis. *Vietti* et al. have proposed that different signaling pathways and biological processes including reactive oxygen species synthesis, inflammatory pathways and endocytosis that are activated in different cell types following exposure to CNTs may together orchestrate proliferation and differentiation of fibroblasts, which, in turn, lead to excessive collagen deposition and fibrosis [71]. The role of myofibroblasts was discussed in *Dong* et al. [72]. The other proposed mechanisms include activation of inflammasome [73], accumulation of CNTs in autophagosomes and disruption of autophagy process [74], activation of TGFb/smad signaling [75], and activation of epithelial-mesenchymal transition. However, essentiality of these key events or molecules in the disease process of lung fibrosis induced by carbon nanotubes is yet to be established.

## Conclusions

The findings of this study expand current understanding of how MWCNTs induce disease. We conclude that signaling through IL-1R1 is a crucial mediator of inflammatory and pro-fibrotic genes 24 h after MWCNT exposure; however, BAL analysis reveal that chronic inflammation is not IL-1R1 dependent, and the formation of fibrotic lesions 28 days after MWCNT exposure is not affected by IL-1R1 deficiency. Transcriptome analysis confirms the transient effects of IL-1R1 deficiency. The results involving STAT6 KO mice showed that STAT6 plays a role in the early neutrophilic response to MWCNT, and significantly suppresses the development of fibrosis 28 days after MWCNT exposure. Transcriptomic analysis identified fibrotic genes that are suppressed at early and later post-exposure timepoints in STAT6 KO mice. Our findings define a mechanism for MWCNT-induced pathology within the context of an AOP that can be used to better understand the biological response to MWCNTs and aid in developing an informed regulatory strategy for these NMs.

## Additional file

Additional file 1: Method for quantifying fibrotic disease area. (PDF 2533 kb)

## Abbreviations

AO: Adverse outcome
AOP: Adverse outcome pathway
BAL: Bronchoalveolar lavage
CNTs: Carbon nanotubes
DAMP: Damage associated molecular pattern
DEGs: Differentially expressed genes
ELISA: Enzyme-linked immunosorbant assay
ENMs: Engineered nanomaterials
GEO: Gene expression omnibus
H&E: Hematoxylin and Eosin
IL: Interleukin
IPA: Ingenuity pathway analysis
KE: Key event
KO: Knock out
MIE: Molecular initiating event
MWCNTs: Multi-walled carbon nanotubes
NALP: NACHT, LRR and PYD domains-containing protein
NM: Nanomaterial
OPN: Osteopontin
STAT: Signal transducer and activator of transcription
TGF: Transforming growth factor
Th: helper T cell
WT: Wild type
