# Supplementary material for: Stat-6 signaling pathway and not Interleukin-1 mediates multi-walled carbon nanotube-induced lung fibrosis in mice: insights from an adverse outcome pathway framework
Source: Part Fibre Toxicol. 2017 Sep 13;14:37. doi: 10.1186/s12989-017-0218-0 (PMC5598059; doi:10.1186/s12989-017-0218-0)
Supplement: Additional file 1: — Method for quantifying fibrotic disease area. (PDF 2533 kb) [file 12989_2017_218_MOESM1_ESM.pdf]

Supplemental Figure

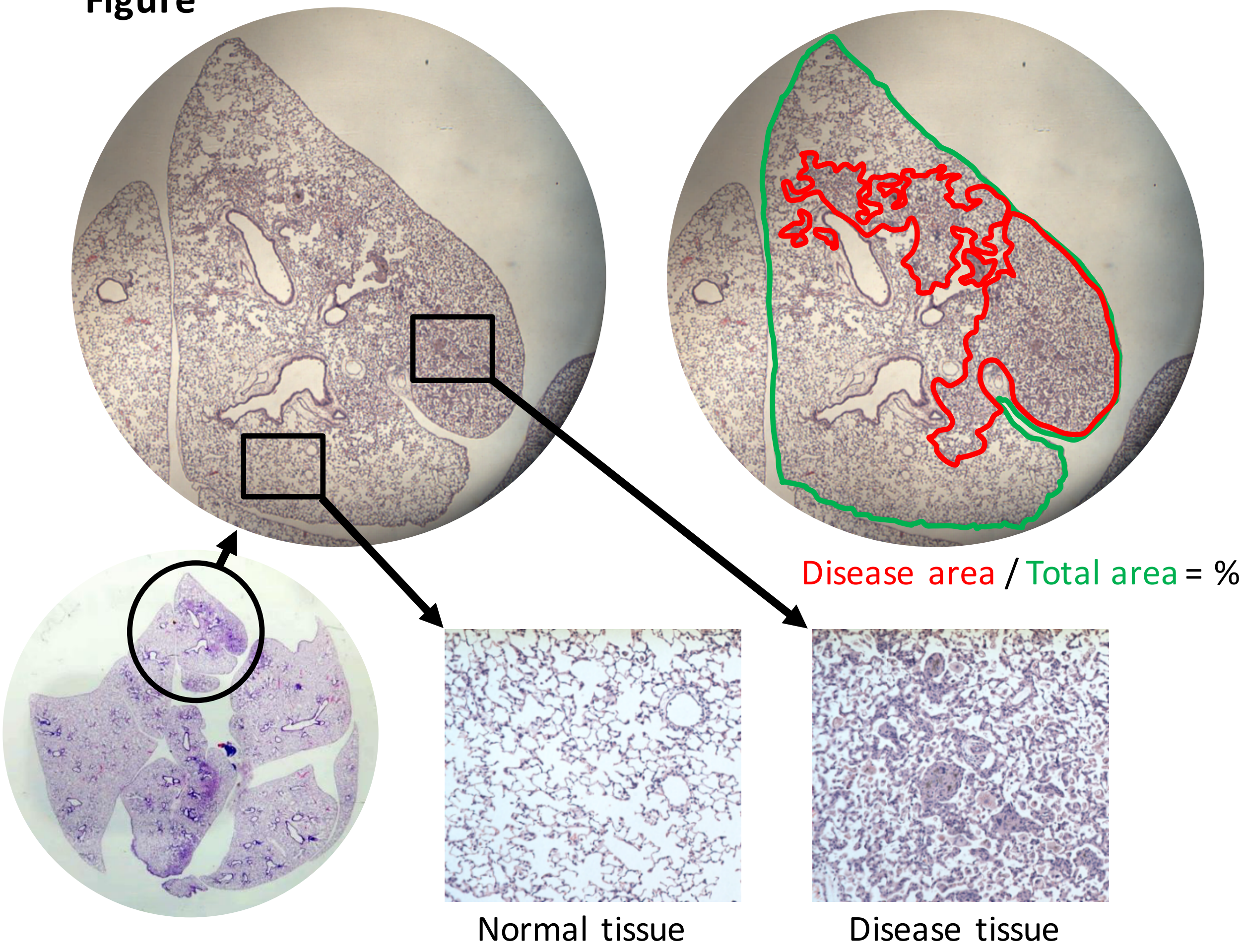

Method for quantifying fibrotic disease area. Lungs were formalin fixed and paraffin embedded such that entire cross sections could be presented on a slide. The entire cross section area was imaged in a series of wide field light microscope pictures. ImageJ software was utilized to trace the total area (green line) and the disease area (red area) based on the identification of thickened epithelium and consolidated airspace. These areas were used to calculate the percent of disease area. All measurements were performed twice by independent researches.
